# S-acylation of SOD1, CCS, and a stable SOD1-CCS heterodimer in human spinal cords from ALS and non-ALS subjects

**DOI:** 10.1038/srep41141

**Published:** 2017-01-25

**Authors:** Sarah E. Antinone, Ghanashyam D. Ghadge, Lyle W. Ostrow, Raymond P. Roos, William N. Green

**Affiliations:** 1 University of Chicago, Department of Neurobiology, Chicago, 60637, USA; 2 University of Chicago, Department of Neurology, Chicago, 60637, USA; 3 Johns Hopkins University, Department of Neurology, Baltimore, 21205, USA

## Abstract

Previously, we found that human Cu, Zn-superoxide dismutase (SOD1) is S-acylated (palmitoylated) *in vitro* and in amyotrophic lateral sclerosis (ALS) mouse models, and that S-acylation increased for ALS-causing SOD1 mutants relative to wild type. Here, we use the acyl resin-assisted capture (acyl-RAC) assay to demonstrate S-acylation of SOD1 in human post-mortem spinal cord homogenates from ALS and non-ALS subjects. Acyl-RAC further revealed that endogenous copper chaperone for SOD1 (CCS) is S-acylated in both human and mouse spinal cords, and *in vitro* in HEK293 cells. SOD1 and CCS formed a highly stable heterodimer in human spinal cord homogenates that was resistant to dissociation by boiling, denaturants, or reducing agents and was not observed *in vitro* unless both SOD1 and CCS were overexpressed. Cysteine mutations that attenuate SOD1 maturation prevented the SOD1-CCS heterodimer formation. The degree of S-acylation was highest for SOD1-CCS heterodimers, intermediate for CCS monomers, and lowest for SOD1 monomers. Given that S-acylation facilitates anchoring of soluble proteins to cell membranes, our findings suggest that S-acylation and membrane localization may play an important role in CCS-mediated SOD1 maturation. Furthermore, the highly stable S-acylated SOD1-CCS heterodimer may serve as a long-lived maturation intermediate in human spinal cord.

Cu, Zn-superoxide dismutase (SOD1) is a ubiquitous homodimeric enzyme that converts superoxide to hydrogen peroxide and oxygen^1^. SOD1 maturation from monomer to a functional enzyme requires zinc binding, copper acquisition, formation of an intramolecular disulfide bond between cysteine (Cys) 57 and Cys 146, and homodimerization^2^,^3^. SOD1 maturation is facilitated by copper chaperone for SOD1 (CCS), which catalyzes the copper acquisition and disulfide oxidation steps^4^,^5^. Like SOD1, CCS is ubiquitously expressed and localizes to the cytosol and intermembrane space of mitochondria^6^,^7^. CCS is a homodimer with a central domain containing high sequence similarity to SOD1^8^. Disulfide and copper transfer from CCS to SOD1 occurs through the formation of a transient heterodimeric complex^2^. The crystal structure of the SOD1-CCS heterodimer shows an intermolecular disulfide bond between Cys 57 of SOD1 and Cys 244 of CCS^9^. In mouse brain tissue, the ratio of SOD1 to CCS was estimated to be 15–30: 1^6^, suggesting that CCS forms many transient interactions with the nascent SOD1 pool in order to activate multiple SOD1 molecules. For each SOD1 maturation event, CCS must localize to the plasma membrane to acquire copper from copper transporter 1 (CTR1)^10^. How CCS is dynamically targeted to membranes is not well characterized.

Amyotrophic lateral sclerosis (ALS) is a neurodegenerative disease characterized by progressive paralysis leading to death from loss of motor neurons in the brain and spinal cord. Mutations in the SOD1 gene cause ~20% of inherited familial ALS (FALS) cases^11^,^12^. Over 150 mutations in SOD1 have been identified to cause FALS. Mice engineered without SOD1 do not develop ALS, and some FALS-causing SOD1 mutations do not alter SOD1 enzymatic activity, thus SOD1-mediated FALS does not appear to result from a loss of SOD1 function. A number of pathological processes have been implicated in mutant SOD1-mediated FALS, including mitochondrial dysfunction, deficient protein quality control, increased ER stress and unfolded protein response, glutamate excitotoxicity, and defects in axonal transport. It is unclear if these abnormalities are pathogenic, or if they are downstream responses to a more primary defect. Mutant SOD1 is prone to improper folding, a consistent feature in SOD1-mediated FALS, suggesting that strategies to maintain the structural stability of SOD1 may represent a therapeutic target (reviewed in ref. 13).

S-acylation (also known as palmitoylation) is a reversible post-translational modification that results in the addition of fatty acids (typically palmitate) to Cys residues. S-acylation regulates protein trafficking, localization to distinct membrane domains, protein stability, and protein-protein interactions. This modification is dynamic, with cycles of S-acylation and de-acylation targeting soluble proteins to and from membranes. Altered S-acylation has been implicated in neurodegenerative diseases such as Huntington’s and Alzheimer’s disease^14^. We previously reported that SOD1 undergoes S-acylation and that FALS-causing SOD1 mutations increase the proportion of S-acylated SOD1 when expressed in cell culture and in ALS mouse models. In addition, we found that S-acylation predominantly occurs on the immature disulfide-reduced form of SOD1 and that S-acylation of the total SOD1 pool is increased under conditions where there is a higher proportion of the immature species^15^. Immature SOD1 monomers are prone to misfolding and aggregation, and are the main component of the insoluble cytoplasmic aggregates detected in SOD1-mediated FALS^16^,^17^,^18^,^19^,^20^.

In this study, we use acyl resin-assisted capture^21^ (acyl-RAC) to quantify SOD1 S-acylation in post-mortem human spinal cord homogenates from ALS and non-ALS subjects. We find that endogenous SOD1 is S-acylated at relatively low levels in human patient samples, with a trend toward increased levels in both sporadic and familial ALS patients relative to non-ALS subjects. Furthermore, CCS is also S-acylated in both human and mouse spinal cord tissues as well as HEK293 cells. Contrary to SOD1, we observed a trend toward decreased CCS S-acylation in ALS patients relative to non-ALS subjects. In addition, we report the presence of a stable and highly S-acylated SOD1-CCS heterodimer complex in human spinal cord tissue that was only observed *in vitro* when both SOD1 and CCS were overexpressed. The heterodimer did not form *in vitro* with CCS or SOD1 cysteine-mutations that attenuate SOD1 maturation. The heterodimer was S-acylated in all samples with a higher proportion being S-acylated than observed for SOD1 and CCS monomers. The heterodimer complex was resistant to heat, SDS, strong denaturants, and reducing agents.

Overall our findings suggest that SOD1, CCS, and SOD1-CCS heterodimers are targeted to membranes by S-acylation, which would facilitate membrane interactions necessary for SOD1 maturation. In addition, the SOD1-CCS heterodimer may represent a long-lived maturation intermediate in human spinal cord.

## Results

### Detection of SOD1 S-acylation by acyl-RAC

In a previous study^15^, we used the acyl-biotin exchange method^22^ (ABE) to assay SOD1 S-acylation. By ABE, we found that the disulfide-reduced form of SOD1 is the predominantly S-acylated species^15^. Subsequently, we discovered that immunoprecipitation of SOD1 does not pull down all disulfide-reduced SOD1, making it difficult to quantitatively assess total SOD1 S-acylation levels (data not shown). In order to assay the S-acylation of the total pool of SOD1 protein, we used the acyl-RAC method, which is similar to ABE but assesses proteins from the whole lysate as opposed to only immunoprecipitated proteins^21^. With this approach, unmodified free cysteines are blocked with the thiol reactive reagent N-ethylmaleimide (NEM) prior to palmitate cleavage with hydroxylamine (NH_2_OH). Removal of palmitate generates free sulfhydryl groups, which are then captured on thiopropyl-sepharose and subsequently eluted and analyzed by SDS-PAGE and Western blotting. In all acyl-RAC experiments, duplicate samples are not treated with NH_2_OH, consequently there is no palmitate cleavage and no sulfhydryl specific pull down with thiopropyl-sepharose.

**Figure 1:**
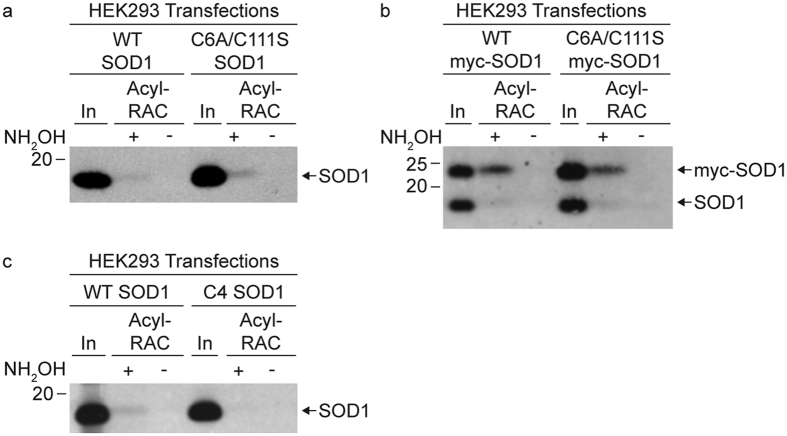
Acyl-RAC detection of SOD1 cysteine mutant S-acylation. HEK293 cells were transiently transfected with (**a**) WT SOD1 or C6A/C111S SOD1, (**b**) myc-tagged WT SOD1 or myc-tagged C6A/C111S SOD1, or with (**c**) WT SOD1 or C6A/C57S/C111S/C146S (C4) SOD1. Equal amounts of total protein lysates were subjected to acyl-RAC analysis. 1% of input (In) protein, 100% of +NH_2_OH acyl-RAC bound protein, and 100% of −NH_2_OH acyl-RAC bound protein were analyzed by Western blotting with an anti-SOD1 antibody.

Our previous analysis using the ABE method showed that mutating both Cys 6 and Cys 111 (C6A/C111S SOD1) prevented the detection of SOD1 S-acylation^15^. In contrast, acyl-RAC analysis of the C6A/C111S SOD1 mutant expressed in HEK293 cells indicated that a portion of the C6A/C111S SOD1 was S-acylated (Fig. 1a). To ensure that we assessed the overexpressed Cys mutant of SOD1 and not endogenous wild type (WT) SOD1, we performed acyl-RAC analysis on myc-tagged versions of WT and C6A/C111S SOD1 from HEK293 cell lysates. The mutant myc-tagged SOD1 could be distinguished from endogenous SOD1 because it runs at a higher molecular weight by SDS-PAGE. S-acylation levels were similar for both WT and C6A/C111S myc-SOD1 by acyl-RAC (Fig. 1b). Human SOD1 lacking all 4 Cys residues (C6A/C57S/C111S/C146S SOD1; referred to as C4 SOD1) expressed in HEK293 cells was not pulled down by acyl-RAC, indicating the assay is specific for Cys S-acylation (Fig. 1c). These results suggest that acyl-RAC is capturing an additional fraction of S-acylated SOD1 that is not recognized by SOD1 antibodies previously used for immunoprecipitation in the ABE assay^15^. This SOD1 fraction appears to be S-acylated on Cys 57 and/or Cys 146, the residues that are disulfide-bonded in the mature SOD1 protein.

### Acyl-RAC analysis of SOD1 and CCS in human spinal cord tissue

**Figure 2:**
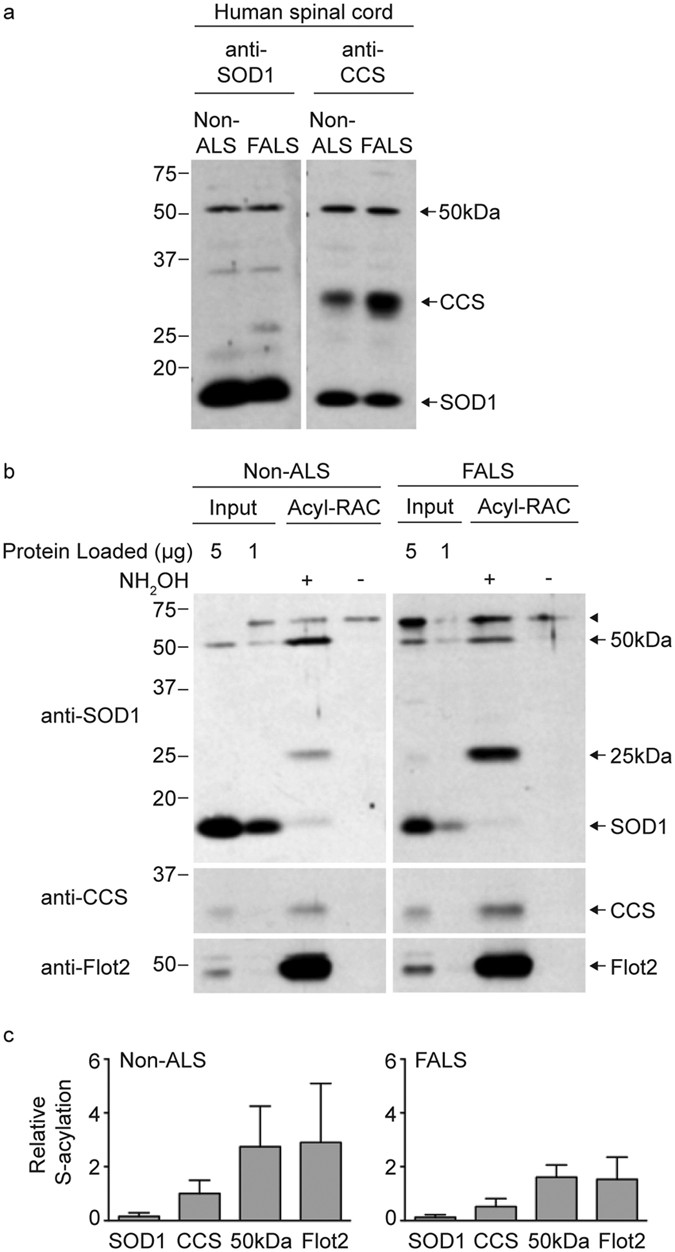
Acyl-RAC detection of SOD1 and CCS S-acylation in human spinal cords from a non-ALS and FALS patient. (**a**) Equal amounts of total protein lysates from human spinal cords were run on SDS-PAGE in duplicate and were analyzed by Western blotting with either an anti-SOD1 antibody or an anti-CCS antibody. (**b**) Equal amounts of total protein lysates from human spinal cords were processed for acyl-RAC. Equal amounts of acyl-RAC input protein, 100% of +NH_2_OH acyl-RAC bound protein, and 100% of −NH_2_OH acyl-RAC bound protein were analyzed by Western blotting first with an anti-SOD1 antibody and subsequently with anti-CCS and anti-flotillin-2 antibodies. (**c**) The densitometry values for +NH_2_OH bound protein signals and input protein signals were corrected for differences in exposure time and input loading. The corrected densitometry values were normalized by dividing the +NH_2_OH bound protein signals by the corresponding input protein signals. Graphs provide average normalized densitometry data and the error bars represent the standard error of the mean (*n* = 4 experiments for the SOD1 monomer, CCS monomer, and 50 kDa band; *n* = 2 experiments for flotillin-2). The non-ALS sample was case 2 and the SOD1 FALS sample was case 9 (see Table 1).

Our results indicate that the acyl-RAC assay provides a more complete assessment of total SOD1 S-acylation than the ABE assay. We therefore used acyl-RAC to quantify SOD1 S-acylation in post-mortem human spinal cord tissues from ALS and non-ALS subjects (Fig. 2 and Table 1). Human spinal cord lysates were first characterized by Western blot with antibodies specific for either SOD1 or CCS. The anti-SOD1 antibody detected SOD1 monomers at 18 kDa and a higher molecular weight band at ~50 kDa. The anti-CCS antibody detected CCS monomers at 34 kDa, SOD1 monomers at 18 kDa, and the same higher molecular weight band at ~50 kDa (Fig. 2a). Cross-reactivity of the CCS antibody for SOD1 is not surprising given the homology between SOD1 and CCS.

Figure 2b shows examples of Western blots from acyl-RAC-assayed lysates of human spinal cord. SOD1 monomers were pulled down in +NH_2_OH treated samples and not in the −NH_2_OH negative control samples, demonstrating that endogenous SOD1 is S-acylated *in vivo*. Acyl-RAC analysis similarly showed that CCS was S-acylated in the human spinal cord tissues. CCS S-acylation was also evident by acyl-RAC in HEK293 cells (Supplemental Fig. S1). The acyl-RAC Western blots were also probed with an antibody against flotillin-2 (Flot2; Fig. 2b), a known S-acylated protein, which served as a positive control throughout this study. Two additional S-acylated anti-SOD1 reactive bands of uncertain identity were detected by acyl-RAC (at ~25 kDa and ~50 kDa). A ~60 kDa (arrowhead) band was also present in all acyl-RAC experiments, but was pulled down in samples that were not treated with NH_2_OH, indicating it is as a non-specific band. The 25 kDa anti-SOD1 reactive band was not visible or appeared as a faint band on Western blots from cell lysates but was prominent after acyl-RAC pulldown, indicating it was highly S-acylated. One possibility is that this is an insoluble complex containing SOD1.

The acyl-RAC analysis allowed us to compare relative levels of S-acylation for SOD1, CCS, and the 50 kDa band to that of flotillin-2. In order to compare the relative S-acylation levels, we normalized the amount of each protein pulled down by acyl-RAC to the amount of that protein present in the input samples used for acyl-RAC. More specifically, densitometry values of +NH_2_OH acyl-RAC bound protein bands were divided by their corresponding input band intensities for the two human patients shown in Fig. 2 (non-ALS case 2 and SOD1 FALS case 9). The densitometry values were adjusted to correct for differences in the amount of input loaded as well as differences in exposure time. Figure 2c shows the corrected average +NH_2_OH bound/input densitometry values for each protein. The flotillin-2 densitometry signals appeared to be saturated in some cases and as a result flotillin-2 S-acylation levels may be underestimated. From this analysis, the rank order of S-acylation levels in human spinal cord was flotillin-2 and the 50 kDa band >CCS monomers > SOD1 monomers. SOD1 S-acylation was relatively low with flotillin-2 S-acylation levels found to be ~20 fold (non-ALS) and ~13 fold (FALS) higher than that of the SOD1 monomer. However, it remains possible that the anti-SOD1 reactive 50 kDa band contains S-acylated SOD1. Similar relative S-acylation trends were seen for each protein band regardless of whether the patient had ALS or not. Detailed comparisons of the S-acylation of SOD1, CCS, and the 50 kDa band in spinal cord tissues from different types of patients (ALS and non-ALS) are made later in the paper (Fig. 5c).

### The 50 kDa band is a heterodimer complex of SOD1 and CCS

**Figure 3:**
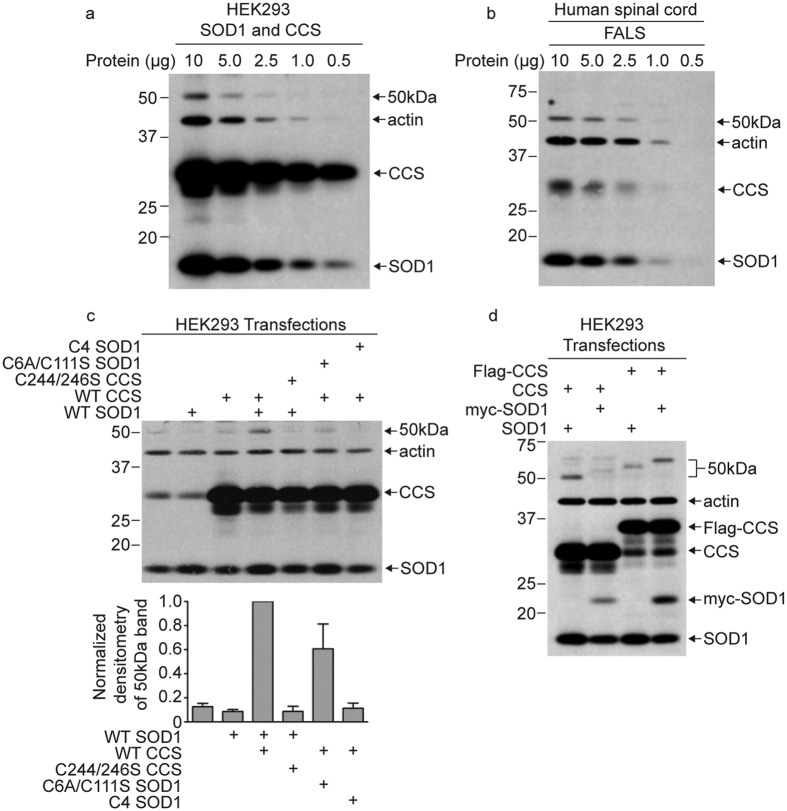
The 50 kDa band is a heterodimer of SOD1 and CCS. (**a**) HEK293 cells were transiently transfected with SOD1 and CCS and decreasing amounts of total protein from transfected cell lysates were analyzed by Western blotting with anti-SOD1, anti-CCS, and anti-actin antibodies. (**b**) Decreasing amounts of total protein from a human spinal cord lysate (FALS case 9) were analyzed by Western blotting with anti-SOD1, anti-CCS, and anti-actin antibodies. (**c**) HEK293 cells were transiently transfected with the indicated WT and mutant versions of SOD1 and CCS. Equal amounts of total protein from transfected cell lysates were analyzed by Western blotting with anti-SOD1, anti-CCS, and anti-actin antibodies. The bar graph provides the average normalized densitometry of the 50 kDa band displayed as a fraction of the group with co-overexpression of WT SOD1 and WT CCS, which is set to one. The error bars represent the standard error of the mean (*n* = 3 or more experiments for each condition). (**d**) HEK293 cells were transiently transfected with the indicated untagged and tagged versions of SOD1 and CCS. Equal amounts of total protein from transfected cell lysates were analyzed by Western blotting with anti-SOD1, anti-CCS, and anti-actin antibodies. An identical experiment with anti-myc and anti-flag Western blot analysis is presented in Supplemental Fig. S3.

The 50 kDa band was recognized on Western blots by both anti-SOD1 and anti-CCS antibodies, suggesting that it could be a heterodimer composed of SOD1 and CCS. In HEK293 cells, the 50 kDa band was not detected by Western blot unless both SOD1 and CCS were overexpressed (Fig. 3c and Supplemental Fig. S1). In Fig. 3a and b, we compare the relative amounts of SOD1, CCS, and the 50 kDa band in HEK293 cells overexpressing SOD1 and CCS with human spinal cord tissue from a FALS patient. By loading decreasing amounts of total protein for Western blot analysis, we found the levels of the 50 kDa band were similar in spinal cord and HEK293 cells, despite there being lower amounts of SOD1 and CCS monomers in spinal cord than in the transfected HEK293 cells.

We next tested whether the 50 kDa band is affected by mutations in SOD1 or CCS that prevent CCS-mediated SOD1 maturation (Fig. 3c). WT and mutant versions of human SOD1 and CCS were expressed in HEK293 cells and lysates were analyzed by Western blot analysis for SOD1 and CCS. The 50 kDa band intensity was strongest when both WT SOD1 and WT CCS were overexpressed. In addition, the 50 kDa band level was decreased in intensity when WT SOD1 was co-expressed with a CCS mutant with Cys residues 244 and 246 changed to serine. The C224/246 S CCS mutant is unable to facilitate copper transfer or disulfide oxidation of SOD1^23^,^24^,^25^. Similarly, co-expression of WT CCS with mutant SOD1 lacking all 4 Cys residues (C4 SOD1) resulted in the loss of the 50 kDa band. Importantly, C4 SOD1 lacks Cys 57, which is involved in the interaction with CCS^9^. Co-expression of WT CCS with C6A/C111S SOD1, which has Cys 57 intact, resulted in less of a decrease of the 50 kDa band than occurred with C4 SOD1 (Fig. 3c). These results indicate that formation of the 50 kDa band is dependent on a functional interaction between SOD1 and CCS.

To further test whether the 50 kDa band is a SOD1-CCS heterodimer, we transiently co-expressed SOD1 and CCS with different tags in HEK293 cells. We used a myc tag for SOD1 and flag tag for CCS, which increased the molecular weight of SOD1 and CCS by distinct amounts. We then tested how the different tags on SOD1 and CCS affected the migration of the 50 kDa band using SDS-PAGE and Western blot analysis. As shown in Fig. 3d, the migration of the 50 kDa band slowed corresponding to shifts in molecular weight based on the presence of each protein tag (Fig. 3d and Supplemental Fig. S3). These results demonstrate that both SOD1 and CCS are present in the 50 kDa band and support the conclusion that the 50 kDa band is a heterodimer complex containing one copy of SOD1 and one copy of CCS.

Like the 50 kDa band present in human spinal cords, the SOD1-CCS heterodimer from HEK293 cell lysates is also found to be S-acylated by acyl-RAC (Supplemental Fig. S1). Whether the S-acylation of the SOD1-CCS heterodimer is due to the S-acylation of SOD1, CCS, or both could not be determined.

### The SOD1 and CCS heterodimer is resistant to denaturation and disulfide reduction

**Figure 4:**
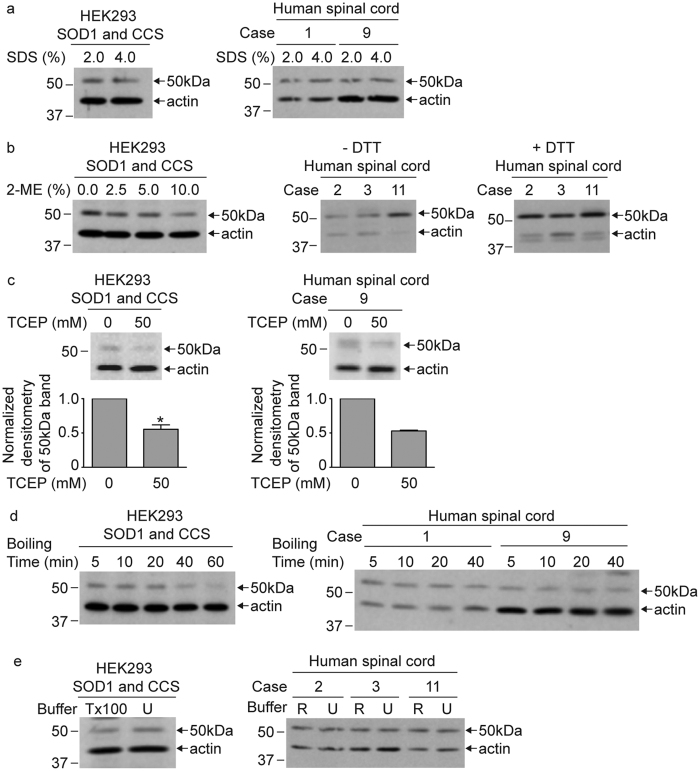
The SOD1 and CCS heterodimer is resistant to denaturing conditions and disulfide reduction. (**a**) Equal amounts of total protein from HEK293 cell lysates transiently co-expressing SOD1 and CCS or human spinal cord lysates were combined with Laemmli buffer containing either 2% SDS or 4% SDS. Each sample was treated with 100 mM DTT and boiled for 5 min. (**b**) Equal amounts of total protein from HEK293 cell lysates transiently co-expressing SOD1 and CCS were combined with Laemmli buffer containing 2% SDS and treated with increasing amounts of 2-mercaptoethanol (2-ME). Equal amounts of total protein from human spinal cord lysates were combined with Laemmli buffer containing 2% SDS and were either treated or not with 100 mM DTT. All samples were boiled for 5 minutes. (**c**) Equal amounts of total protein from HEK293 cell lysates transiently co-expressing SOD1 and CCS or a human spinal cord lysate (FALS case 9) were combined with Laemmli buffer containing 2% SDS and were either treated or not with 50 mM TCEP. All samples were boiled for 5 minutes. The bar graphs provide the average normalized densitometry of the 50 kDa band displayed as a fraction of the untreated samples. The error bars represent the standard error of the mean (*n* = 3 experiments for HEK293 cells, *P < 0.05; *n* = 2 experiments for human spinal cord). (**d**) Equal amounts of total protein from HEK293 cell lysates transiently co-expressing SOD1 and CCS or human spinal cord lysates were combined with Laemmli buffer containing 2% SDS and 100 mM DTT. Samples were subsequently boiled for the indicated time points. (**e**) HEK293 cells transiently co-expressing SOD1 and CCS were solubilized in either lysis buffer containing 1% Triton X-100 (Tx100) or 4 M urea and treated with 100 mM DTT. Human spinal cord lysates were solubilized in either RIPA buffer (R) or lysis buffer containing 6 M urea (U) and treated with 100 mM DTT. Samples were boiled for 5 min, with the exception of the urea containing samples. All samples were subjected to standard Western blot analysis with anti-SOD1, anti-CCS, and anti-actin antibodies. Full-length images of Western blots are presented in Supplemental Fig. S4.

The 50 kDa heterodimeric complex between SOD1 and CCS was detected in HEK293 and human spinal cords lysates after boiling in 2% SDS and 100 mM DTT (Figs 2 and 3). The 50 kDa band remained detectable in lysates despite increasing the SDS concentration in Laemmli buffer to 4% (Fig. 4a) or 2-mercaptoethanol up to 10% (Fig. 4b). Similarly, the presence of 100 mM DTT in human spinal cord lysates did not decrease the amount of the 50 kDa band (Fig. 4b). Treatment with 50 mM TCEP, which is a stronger reducing agent than DTT, caused a 50% decrease in the normalized intensity of the 50 kDa band for both HEK293 and human spinal cord lysates (Fig. 4c). The decrease in the levels of the 50 kDa band by disulfide reduction suggests a disulfide linkage between SOD1 and CCS in the heterodimer complex. We also tested harsher denaturing conditions by increasing the boiling time for up to 1 h in Laemmli buffer and 100 mM DTT before Western blot analysis. Despite some decrease in band intensity, the 50 kDa band could still be detected in HEK293 lysates. Similarly, the 50 kDa band remained in human spinal cord lysates subjected to 40 min of boiling in Laemmli buffer and 100 mM DTT (Fig. 4d). The 50 kDa heterodimer complex also remained detectable following solubilization in buffer containing 4 M urea (Fig. 4e). The SOD1-CCS heterodimer present in our HEK293 lysates and human spinal cord tissue is thus resistant to strong denaturation and disulfide reduction.

### SOD1 and CCS S-acylation in ALS versus non-ALS human spinal cord tissues

**Figure 5:**
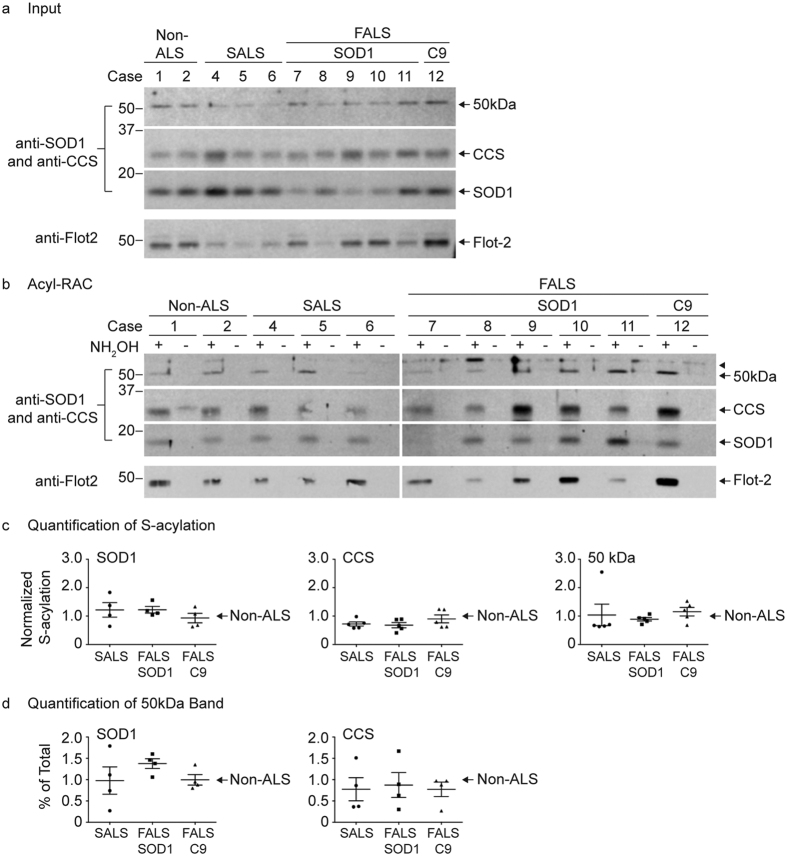
Quantitative acyl-RAC analysis of SOD1 and CCS S-acylation in human spinal cords. (**a**) Equal amounts of acyl-RAC input protein from human spinal cord lysates were analyzed by Western blotting with anti-CCS (10 μg total protein loaded) and anti-SOD1 (5 μg total protein loaded) antibodies. The anti-CCS Western blot was subsequently stripped and analyzed by Western blotting with an anti-flotillin-2 antibody (10 μg total protein loaded). (**b**) Equal amounts of total protein lysates from human spinal cords were processed for acyl-RAC. 100% of +NH_2_OH acyl-RAC bound protein and 100% of −NH_2_OH acyl-RAC bound protein were analyzed by Western blotting with anti-SOD1 and anti-CCS antibodies. The same Western blots were subsequently stripped and analyzed by Western blotting with an anti-flotillin-2 antibody. (**c**) The densitometry values were determined by dividing the +NH_2_OH acyl-RAC bound protein signals by the corresponding input protein signals. The normalized densitometry values were then averaged for each group and all groups were normalized to the non-ALS control average per experiment. Normalization was only done for results within the same experiment in which all samples were processed in parallel. Graphs provide average normalized densitometry data from multiple experiments displayed as a fraction of the non-ALS control group, which is set to one. The horizontal lines represent the mean relative S-acylation level for each group and the error bars represent the standard error of the mean (*n* = 4 experiments for the SOD1 monomer; *n* = 5 experiments for the CCS monomer and 50 kDa band). (**d**) Densitometry analysis was done to determine the ratio of the 50 kDa band as a percentage of total SOD1 or total CCS (50 kDa band densitometry values divided by the sum of the SOD1 or CCS monomer and 50 kDa band densitometry values). Percent of total ratios were then averaged for each group and all groups were normalized to the non-ALS control average per experiment. Graphs provide average percent of total values displayed as a fraction of the non-ALS control group, which is set to one. The horizontal lines represent the mean relative percent of total for each group and the error bars represent the standard error of the mean (*n* = 4 experiments). Full-length images of Western blots are presented in Supplemental Fig. S5.

In order to test whether SOD1, CCS, and 50 kDa band S-acylation changes with ALS, we performed acyl-RAC analysis on post-mortem human spinal cord tissues from 12 human patients (Table 1). Monomers of SOD1 and CCS and the 50 kDa SOD1-CCS heterodimer were detected as S-acylated by acyl-RAC in all subjects. The levels of S-acylation for each protein band were determined by normalizing the amount of +NH_2_OH acyl-RAC bound protein detected on the acyl-RAC Western blots (Fig. 5b) to the amount of that same protein present on the corresponding input Western blots (Fig. 5a), in which equal amounts of total protein were loaded onto the gel for each sample. The values were averaged for each group and all groups were normalized to the non-ALS control average per experiment (Fig. 5c). Different exposure times were necessary for the SOD1 monomer, CCS monomer, and 50 kDa band to obtain results within the linear range for the quantification of S-acylation. Each patient sample was treated identically (same exposure times) within each experiment. Quantification of S-acylation from multiple acyl-RAC experiments revealed a trend in which SOD1 monomer S-acylation levels were higher for the mutant SOD1 FALS and sporadic ALS (SALS) patient samples relative to non-ALS patient samples. In addition, CCS monomer S-acylation levels were lower for mutant SOD1 FALS and SALS patient samples relative to non-ALS patient samples. However, statistical analysis of the quantification results showed no significant differences for S-acylation levels of the SOD1 monomer, CCS monomer, and 50 kDa bands between non-ALS and ALS disease groups. From the densitometry analysis, the ratio of the 50 kDa band as a percentage of total SOD1 was increased for SOD1 FALS patients compared to non-ALS patients but this difference was not statistically significant (Fig. 5d).

### SOD1 and CCS S-acylation in mouse spinal cords by acyl-RAC

Next we used acyl-RAC to examine SOD1 and CCS S-acylation levels in transgenic mouse models of ALS. Mouse spinal cord lysates from two G93A SOD1 mice (5.5 months of age) were first examined by Western blot analysis with either the anti-SOD1 or anti-CCS antibody (Supplemental Fig. S2). The anti-SOD1 antibody detected SOD1 monomers at 16 kDa (mouse) and 18 kDa (human) as well as a higher molecular weight band at ~40 kDa. The anti-CCS antibody detected CCS monomers at 34 kDa, SOD1 monomers at 16 kDa and 18 kDa, and two higher molecular weight bands at ~40 kDa and ~50 kDa. Because the anti-SOD1 antibody does not cross-react with CCS, the 40 kDa band likely contains at least one copy of SOD1 and may also contain one copy of CCS. Complicating the interpretation is the significant cross-reactivity of the anti-CCS antibody to SOD1 monomers, thus it is possible that the 40 kDa band does not include CCS. While the anti-SOD1 antibody did not significantly stain the 50 kDa band, it remains possible this band is a CCS-SOD1 heterodimer in which the anti-SOD1 antibody epitope is masked, perhaps by the presence of mouse CCS. Because of these complications, we could not determine whether the higher molecular weight bands contain only one or both proteins.

**Figure 6:**
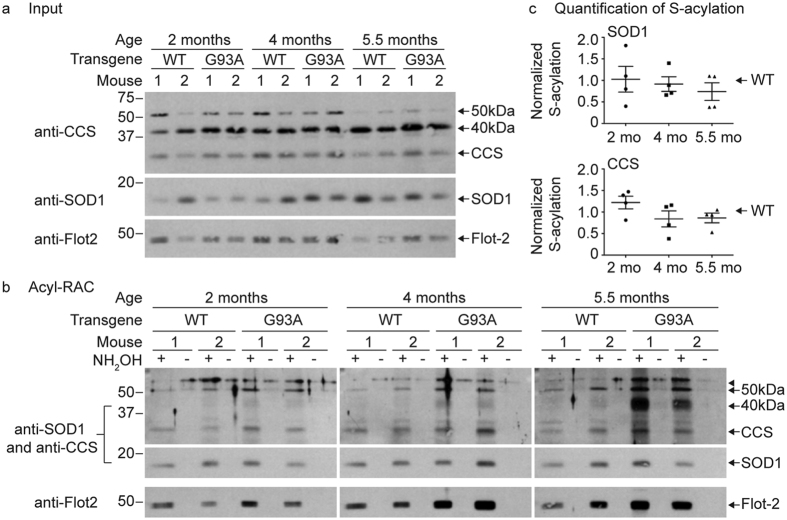
Quantitative acyl-RAC analysis of SOD1 and CCS S-acylation in mouse spinal cords. (**a**) Equal amounts of acyl-RAC input protein from spinal cords harvested from human WT SOD1 or G93A SOD1 transgenic mice were analyzed by Western blotting with an anti-flotillin-2 antibody (1 μg total protein loaded), an anti-CCS antibody (5 μg total protein loaded), or an anti-SOD1 antibody (0.5 μg total protein loaded). (**b**) Equal amounts of total protein from spinal cords harvested from human WT SOD1 and G93A SOD1 transgenic mice were subjected to acyl-RAC and 100% of +NH_2_OH acyl-RAC bound protein and 100% of −NH_2_OH acyl-RAC bound protein were analyzed by Western blotting with anti-SOD1 and anti-CCS antibodies. The same Western blots were subsequently stripped and analyzed by Western blotting with an anti-flotillin-2 antibody. (**c**) The densitometry values were determined by dividing the +NH_2_OH bound protein signals by the corresponding input protein signals. The normalized densitometry values were then averaged for each age group and G93A average values were normalized to the corresponding WT SOD1 average values per experiment. Normalization was only done for results within the same experiment in which all samples were processed in parallel. Graphs provide average normalized densitometry data from multiple experiments displayed as a fraction of WT SOD1, which is set to one for each experiment. The horizontal lines represent the mean relative S-acylation level for each group and the error bars represent the standard error of the mean (*n* = 4 experiments, two mice spinal cords were examined per condition for each experiment). Full-length images of Western blots are presented in Supplemental Fig. S6.

To examine whether SOD1 S-acylation levels change over the course of disease progression in mutant SOD1 transgenic mice, we used acyl-RAC to purify S-acylated proteins from spinal cords harvested from either human WT SOD1 or G93A SOD1 transgenic mice at three different ages: 2 months (mo) (pre-symptomatic), 4 months (symptom onset), and 5.5 months (end stage). Spinal cords from two mice were examined per condition per experiment. As shown in Fig. 6b, S-acylated monomers of SOD1 and CCS were detected at all ages in both the human WT SOD1 and G93A mutant SOD1 mice (Fig. 6b). The levels of SOD1 and CCS S-acylation in the mouse spinal cords were determined by normalizing the amount of +NH_2_OH bound protein detected on the acyl-RAC Western blots (Fig. 6b) to the amount of that same protein present on the corresponding input Western blots (Fig. 6a), in which equal amounts of total protein were loaded onto the gel for each sample. The values were averaged for each age group and G93A average values were normalized to the corresponding WT SOD1 average values per experiment (Fig. 6c). Different exposure times were necessary for SOD1 and CCS in order to obtain results within the linear range for the quantification of S-acylation. Each sample was treated identically (same exposure times) within each experiment. Quantification of S-acylation levels for both SOD1 and CCS monomers revealed no statistically significant differences between WT and G93A SOD1 mice at any age. In addition, there were no statistically significant differences in the S-acylation levels of the SOD1 or CCS monomers over the course of disease in the SOD1 G93A spinal cords.

The transgenic SOD1 mouse models used in this study have extremely high levels of SOD1 protein expression (~20 fold increase over non-transgenic controls) and as a result have increased steady state levels of disulfide-reduced SOD1 relative to control^17^. Importantly, we find disulfide-reduced SOD1 to be the predominantly S-acylated species^15^. Thus, the degree of SOD1 S-acylation in transgenic mouse models is likely to be artificially high and may not reflect biologically relevant levels of this post-translational modification. Consistent with this, we find the level of SOD1 monomer S-acylation in transgenic SOD1 mice spinal cords to be significantly higher by acyl-RAC than that of endogenous SOD1 from human spinal cord tissues, which require longer Western blot exposures in order to detect. It is possible that high levels of SOD1 S-acylation for both WT and G93A SOD1 in the transgenic mice could obscure subtle changes in SOD1 monomer S-acylation that may be relevant to ALS.

The 50 kDa band was present in the input of all samples and was detected as S-acylated by acyl-RAC in both WT and G93A SOD1 mice with no obvious trend in S-acylation levels. In contrast, the 40 kDa band was present in inputs of all samples but S-acylation was only faintly detected for WT and G93A SOD1 at 2 months of age. Importantly, the 40 kDa band S-acylation levels dramatically increased with age and disease symptoms in the G93A SOD1 mice but not the age-matched WT SOD1 mice (Fig. 6b). The 40 kDa band is recognized by our anti-SOD1 antibody, which does not cross-react with CCS, suggesting that SOD1 is likely to be present in this band. Thus, SOD1 present in the 40 kDa band may become more highly S-acylated during ALS-like disease relative to earlier stages when the mice are pre-symptomatic.

## Discussion

Our previous experiments examining SOD1 S-acylation were performed using transfected cells or transgenic mice in which SOD1 was overexpressed. SOD1 overexpression increases the disulfide-reduced SOD1 species, which is the predominantly S-acylated form of the protein. As a result, overexpression of SOD1 leads to increased levels of SOD1 S-acylation relative to when the protein is at endogenous levels^15^. In this study, we have addressed whether SOD1 endogenous to human spinal cords is S-acylated and whether the levels of this modification are altered in ALS. We used the acyl-RAC method to assay S-acylation, given that anti-SOD1 antibody immunoprecipitation fails to capture a fraction of S-acylated SOD1 (Fig. 1). The acyl-RAC method has equal access to all proteins in the cell lysate. However, it is only a measure of whether a protein of interest is S-acylated and cannot distinguish whether S-acylation occurs at one or more than one site. In this way, acyl-RAC is less quantitative than other methods. Nonetheless, acyl-RAC allowed for a quantitative comparison of S-acylation levels between ALS and non-ALS patient groups (Fig. 5). The acyl-RAC analysis demonstrated that endogenous SOD1 in human spinal cord is S-acylated. SOD1 monomer S-acylation was detected in all samples and appeared to increase in the FALS and SALS patients relative to non-ALS patients, although the increase was not statistically significant. We also found that endogenous CCS was S-acylated using the same acyl-RAC analysis of human spinal cord tissues. CCS S-acylation, in contrast to SOD1, appeared to be decreased in ALS relative non-ALS patients, although again the decrease was not statistically significant.

During our acyl-RAC analysis, we discovered the presence of stabilized heterodimers between SOD1 and CCS in human spinal cord lysates. Based on its 50 kDa size, the stabilized heterodimer has a 1:1 SOD1:CCS stoichiometry. The degree of S-acylation of the heterodimer was relatively high. However, we were unable to test whether S-acylation of SOD1 is responsible for the S-acylation of the complex because the non-S-acylated C4 mutant of SOD1 does not form SOD1-CCS heterodimers. From our acyl-RAC analysis we determined that S-acylation of SOD1 was relatively lower than that of CCS but both were significantly less S-acylated than that of flotillin-2. The S-acylation levels of SOD1-CCS heterodimers, in contrast, were closer to that of flotillin-2. Thus, SOD1-CCS heterodimers appear to have higher amounts of S-acylated SOD1 and/or CCS than their respective monomer population. This suggests that S-acylated forms of these proteins may have a higher propensity to form this heterodimer. We also detected an increase in the levels of SOD1-CCS heterodimers for FALS patients relative to non-ALS patients (Fig. 5d). This suggests that mutant SOD1 may form more of this stable complex with CCS, as has been established for other ALS-causing SOD1 mutants^26^.

By several criteria, we found that SOD1-CCS heterodimers were difficult to disrupt. Specifically, the 50 kDa band was resistant to SDS solubilization, reducing agents, urea buffer and even extensive boiling (Fig. 4), similar to the homodimer that forms with inducible nitric oxide synthase (iNOS)^27^. For the heterodimer to be so resistant to dissociation, it is likely that a disulfide bond is covalently linking SOD1 and CCS. Consistent with disulfide-linkage, TCEP treatment resulted in the loss of approximately 50% of the 50 kDa band. One possible explanation for why we cannot disrupt all of the SOD1-CCS heterodimer with disulfide reduction is that the disulfide bond could be buried in the protein structure making it inaccessible to reducing agents. During SOD1 maturation, CCS catalyzes the formation of the SOD1 intramolecular disulfide bond through an intermolecular thiol-disulfide exchange reaction^2^,^5^. This disulfide exchange involves the formation of an intermolecular disulfide bond between Cys 57 of SOD1 and Cys 244 of CCS^9^. The disulfide-bonded SOD1-CCS heterodimer is thought to be transient because it is not observed for WT SOD1 and has only been detected with a mutant version of SOD1 (H48F) that cannot bind copper^9^,^26^,^28^,^29^. Following disulfide transfer from CCS to SOD1, SOD1 is thought to change to a form that favors homodimerization and dissociate from CCS^5^. Loss of SOD1 zinc binding disrupts the formation and disassociation of the heterodimer^29^. Whether the SOD1-CCS heterodimer that we observe is a longer-lived version of the functional SOD1 maturation intermediate is not clear at this time. Another possibility is that the heterodimer we observe results from a faulty interaction between SOD1 and CCS that is deficient for dissociation, similar to what occurs with metal-depleted mutants of SOD1. A third possibility is that the SOD1-CCS heterodimer disulfide bond does not occur *in vivo* and instead occurs upon solubilization when the conditions become more oxidative and conducive to disulfide bond formation. However, treating HEK293 cells that were overexpressing SOD1 and CCS with NEM prior to solubilization did not prevent the formation of the 50 kDa band, arguing against this possibility (data not shown). Regardless of the functional significance, these findings suggest that there are special conditions present in human spinal cord, but not HEK293 cells, that promote highly S-acylated, difficult to disrupt SOD1-CCS heterodimers to form.

In mouse spinal cords, we were unable to identify whether the higher molecular weight bands we observe correlate with the SOD1-CCS heterodimers present in human spinal cords. The anti-CCS antibody recognized both higher molecular weight bands, while the anti-SOD1 antibody recognized the 40 kDa and not the 50 kDa band (Supplemental Fig. S2). These findings suggest that the 40 kDa band could be a heterodimer complex of human SOD1 and mouse CCS that migrates faster in SDS-PAGE than the equivalent complex in human spinal cords. However, it is possible that a lack of staining of the 50 kDa band by the anti-SOD1 antibody is caused by the interaction with CCS blocking or reducing antibody access to the SOD1 epitope. Thus, it remains possible that the 50 kDa band detected by the anti-CCS antibody could be a heterodimer complex of human SOD1 and mouse CCS or it could be some other CCS containing complex such as an insoluble CCS homodimer that has not yet been described. It is also possible the 40 kDa band represents an insoluble homodimer of SOD1 previously observed in G93A SOD1 mice spinal cords^30^,^31^. Importantly, the S-acylation of the 40 kDa complex clearly increases as ALS progresses from early to late stages of the disease in G93A SOD1 mice. Despite the fact that we have not been able to determine the identity of the 40 kDa and 50 kDa bands from mouse spinal cords, our findings indicate that dimers of SOD1 and/or CCS have increased S-acylation as ALS progresses. A similar 50 kDa band was previously observed in spinal cords from transgenic mice co-expressing human WT CCS and human G93A SOD1, which was decreased in age-matched G93A SOD1 mice^32^,^33^. The CCS/G93A SOD1 mice have accelerated disease relative to G93A SOD1 mice^32^ consistent with an altered CCS-SOD1 interaction increasing FALS pathology.

SOD1 is predominantly cytosolic with a fraction of the protein localizing to different organelle membranes including the membranes of mitochondria^7^ and the ER-Golgi pathway^34^,^35^. Mitochondrial localization only occurs for disulfide-reduced apo-SOD1^36^, and is dependent on CCS^37^. Once imported, SOD1 maturation is catalyzed by mitochondrial localized CCS^36^. Thus, one possibility is that S-acylation targets immature SOD1 and/or CCS to intracellular organelles in order for them to function at those sites. It is established that CCS goes to membranes where the copper transporter, CTR1, loads copper on to CCS prior to SOD1 folding. A lipid-binding interface has been identified for CCS that was proposed to promote CTR1-CCS and CCS-SOD1 copper exchange at membranes^10^. S-acylation of CCS and SOD1 would enhance their ability to interact with membranes, and could do so in a dynamic and regulated manner. For CCS and SOD1 to be S-acylated they first must bind and interact with zinc-finger, palmitoyl-transferases (PATs), which are integral membrane proteins that mediate S-acylation. Thus, interactions with PATs and subsequent S-acylation could position CCS and SOD1 to allow for CCS copper acquisition and efficient distribution to SOD1. Our discovery that SOD1-CCS heterodimers are highly S-acylated and their formation is blocked by mutations of functional cysteine residues in CCS and SOD1 further supports the hypothesis that S-acylation targets nascent SOD1 and CCS to membranes in order to enhance SOD1 maturation. One possible role for S-acylated SOD1-CCS heterodimers is to recruit SOD1 for more rapid folding when needed, for instance in areas where the superoxide concentration is highest.

## Methods

### Antibodies and reagents

The following primary antibodies were used: rabbit anti-SOD1 (Enzo Life Sciences), rabbit anti-CCS (Protein Tech), rabbit anti-actin (Sigma Aldrich), and rabbit anti-flotillin-2, which was a gift from Gopal Thinakaran and was previously described^38^. All primary antibodies were detected with a horseradish peroxidase (HRP) conjugated goat anti-rabbit IgG (Jackson ImmunoResearch). The following reagents were used: Hydroxylamine (NH_2_OH), Thiopropyl-Sepharose 6B (Sigma Aldrich), and N-ethylmaleimide (NEM) (Calbiochem).

### DNA constructs

Construction of WT SOD1 plasmid was previously described^39^. Dr. Valeria Culotta (Johns Hopkins University) provided pJP001 plasmids encoding human CCS (pJP001-hCCS) and mutant C244/246 S CCS. The WT myc-SOD1 encoding plasmid was generated by PCR amplification of SOD1 open reading frame (ORF) from WT SOD1 plasmid using forward primer 5′CCGGAATTCATGGCGACGAAGGCCGTGTGCGTG3′ and reverse primer 5′CCCAAGCTTTTGGGCGATCCCAATTACACC3′ followed by cloning into EcoRI and HindIII sites of the pcDNA3.1/myc-His(−) A vector. Plasmid C6A/C111S myc-SOD1 was generated in two steps. First, Cys at position 111 of WT myc-SOD1 was mutated to serine using site-directed mutagenesis. Second, the mutation of Cys at position 6 to serine was introduced by PCR amplification of SOD1 ORF using forward primer 5′CTAGCTAGCATGGCGACGAAGGCCGTGGCCGTG3′ and reverse primer 5′CCCAAGCTTTTGGGCGATCCCAATTACACC3′ followed by inserting the PCR fragment into NheI and HindIII sites of the vector pcDNA3.1/myc-His (−) A. C6A/C111S SOD1 expression plasmid was constructed by PCR amplification of the SOD1 ORF from the C6A/C111S myc-SOD1 encoding plasmid using forward primer 5′CTAGCTAGCATGGCGACGAAGGCCGTGGCCGTG3′ and reverse primer 5′CCGCTCGAGTTATTGGGCGATCCCAATTACACC3′ followed by cloning into NheI and XhoI restriction enzyme sites of the pcDNA3.1(+) vector. The plasmid encoding C6A/C57S/C111S/C146S (C4 SOD1) SOD1, with mutation of all four cysteines was generated by site-directed mutagenesis using C6A/C111S SOD1 template and primers 5′GATAATACAGCAGGCTCTACCAGTGCAGG3′ and 5′GGAAGTCGTTTGGCTTCTGGTGTAATTGGG3′. Flag-CCS was generated by cloning the EcoRI-PmeI fragment of pJP001-hCCS into EcoRI and EcoRV sites of the p3xFlag-CMV-10 vector (Sigma Aldrich).

### Cell culture and transfections

HEK293 cells were maintained in Dulbecco’s Modified Eagle Medium (DMEM) with 10% calf serum and 2% penicillin-streptomycin. HEK293 cells in 60-mm or 100-mm culture dishes were transfected at 60–80% confluence with 2–4 μg DNA using a calcium phosphate protocol for 3–5 h and then changed to normal media.

### Human Cases

The human patient samples included in this study were from five FALS mutant SOD1 cases (N139K, V87A, and three with A4V mutations), one non-SOD1 FALS case with a C9orf72 repeat expansion mutation (C9), three SALS cases (negative for SOD1 and C9orf72 mutations), and three non-ALS cases (case 1 was diagnosed with congestive heart failure, hypertension, diabetes and renal failure; case 2 had a prior history of polio; case 3 was diagnosed with Lewy body dementia and peripheral neuropathy). The Target ALS Multicenter Human Postmortem Tissue Core provided Cases 2–11. Cases 1 and 12 were obtained at the University of Chicago. All autopsy tissue samples from patients were analyzed in five separate acyl-RAC experiments except cases 1 and 3, which were analyzed in 3 and 4 experiments respectively. All methods were carried out in accordance with relevant guidelines and regulations set by the Institutional Review Board (IRB) of the University of Chicago where all experiments were done. IRB approval was not necessary with respect to human autopsy samples because the samples did not constitute research with human subjects as samples were collected from deceased individuals and the definition of a human subject specifically references living individuals. However, subjects enrolled in the Target ALS Postmortem Tissue Core signed written, informed consent. In addition, written, informed consent for all autopsies was obtained from next-of-kin after death, and a HIPAA Form 5 exemption was granted to access patient information for decedents who were not enrolled in the Target ALS Core pre-mortem.

### Preparation of spinal cord tissue

Human spinal cord tissue or spinal cords harvested from transgenic mice expressing either human G93A SOD1 (mean survival 157 +/− 1.8 days) or human WT SOD1 (age matched to G93A SOD1 mice) were processed as follows. Ten percent homogenates (weight/volume) in RIPA buffer containing protease inhibitors and 10 mM NEM were prepared from spinal cords using Kontes Microtube Pellets Pestle with motorized homogenizer (A. Daigger and Company). Homogenates were incubated on ice for 30 min and supernatants were collected following centrifugation (8,000 rpm, 4 °C, 10 min) and were subsequently processed for acyl-RAC analysis. All experimental protocols were approved by the Institutional Animal Care and Use Committee (IACUC) at the University of Chicago. All methods were carried out in accordance with IACUC guidelines.

### Acyl-RAC

Acyl-RAC was performed as previously described^21^,^40^ with a few modifications. HEK293 cell lysates or spinal cord homogenates were solubilized in lysis buffer (LB: 150 mM NaCl, 50 mM Tris, 5 mM EDTA, pH 7.4) containing 0.2% Triton-X 100, protease inhibitors, and 10 mM NEM. Supernatants were then collected following centrifugation to remove particulates and insoluble proteins (12,000 rpm, 4 °C, 10 min). Solubilized proteins were concentrated by ultrafiltration on centrifugal filter units with a membrane molecular weight limit of 3 kDa (4,000 g, 30 min)(Millipore). Each concentrated sample was diluted up to 1 ml in LB containing 0.2% Triton-X 100, 10 mM NEM and protease inhibitors and incubated for 20 min at room temperature (RT). Samples were then kept on ice at 4 °C overnight. The following day, NEM was removed by three rounds of ultrafiltration as described above, except that prior to the third and final iteration, binding buffer (BB: 100 mM Hepes, 1 mM EDTA, pH 7.4) with 0.1% SDS was used as the diluent. Following ultrafiltration, the concentrated 250 μl sample was transferred out of the filtration unit along with a 250 μl wash of the unit using BB with 4% SDS. The samples were diluted up to 1 ml with BB to give a final SDS concentration of ~1%. Total protein was quantified following ultrafiltration steps using the BCA assay (Pierce) and BSA standards. Equal amounts of total protein (1–10 μg) were combined with Laemmli buffer to assess input protein levels. The remaining thiol-blocked protein samples were then absorbed to thiopropyl-Sepharose 6B (Sigma Aldrich) either in the presence or absence of NH_2_OH. For acyl-RAC pulldown reactions, equal amounts of total protein (300–500 μg) were mixed with either 150 μl of 2 M NH_2_OH pH 7.2–7.4 (+NH_2_OH) or 150 μl of 0.8 M NaCl (−NH_2_OH) and then added to 100 μl of resin, pre-equilibrated with BB containing 1% SDS. Following rotation at RT for 2 h, unbound protein was removed and the resin was subjected to five washes with 1 ml BB containing 1% SDS. Finally, bound protein was eluted by incubating the washed resin with 100 μl BB containing 1% SDS and 50 mM DTT for 20 min at RT. The eluted protein was separated from the resin by two rounds of centrifugation (8,000 rpm, 1 min) and combined with Laemmli buffer for quantitative western blot analysis.

### Western Blot Analysis

HEK293 cell lysates, human or mouse spinal cord homogenates, or acyl-RAC treated proteins were mixed with Laemmli sample buffer and were separated by SDS-PAGE with 12% polyacrylamide gels and transferred to nitrocellulose membranes. SDS-PAGE was performed under reducing conditions unless specified otherwise. Samples were heated at 100 °C for 5 min prior to SDS-PAGE unless specified otherwise. The following antibody dilutions were used: anti-SOD1 (1:2,000), anti-CCS (1:1,000), anti-actin (1:3,000), and anti-flotillin-2 (1: 20,000). Primary antibody incubations were done for 1 h to overnight (4 °C). All Western blots were subsequently incubated with HRP-conjugated goat anti-rabbit IgG (1:20,000) for 1–2 h and protein bands were detected with luminol-coumaric acid-H_2_O_2_ chemiluminescence. Quantification of band intensity was done using Image J software (NIH).

### Quantification of S-acylation

To determine the relative levels of protein S-acylation between samples (Figs 5 and 6), the +NH_2_OH bound acyl-RAC protein signals were divided by the corresponding input protein signals. The normalized densitometry values were then averaged for each group and all groups were normalized to the non-ALS control average per experiment. The same approach was taken to determine the relative S-acylation levels between SOD1, CCS, 50 kDa band, and flotillin-2 from human spinal cord (Fig. 2), except the densitometry values were adjusted to correct for differences in the amount of input loaded (multiplied values to be equivalent to loading 5ug total protein) as well as differences in exposure time (divided values to be equivalent to a 1 min exposure time).

### Statistical Analysis

One-way ANOVA variance analysis with Newman-Keuls multiple comparison tests were used to calculate statistical significance of spinal cord experiments. Paired t-test was performed to determine the statistical significance of the HEK293 cell experiment with or without TCEP treatment.

## Additional Information

**How to cite this article**: Antinone, S. E. *et al*. S-acylation of SOD1, CCS, and a stable SOD1-CCS heterodimer in human spinal cords from ALS and non-ALS subjects. *Sci. Rep.* **7**, 41141; doi: 10.1038/srep41141 (2017).

**Publisher's note:** Springer Nature remains neutral with regard to jurisdictional claims in published maps and institutional affiliations.

## Supplementary Material

Supplementary Information

## Figures and Tables

**Table 1:** Human Subjects.

Case	Sex	Diagnosis	ALS Causing Mutation	Age at Death	Disease Duration (mo)	Spinal Cord Region
1	F	Non-ALS	N/A	68	N/A	cervical
2	F	Non-ALS	N/A	90	N/A	cervical
3	M	Non-ALS	N/A	80	N/A	cervical
4	F	SALS	None	68	70	cervical
5	F	SALS	None	70	36	lumbar
6	F	SALS	None	56	50	lumbar
7	M	FALS	V87A SOD1	58	60	cervical
8	M	FALS	A4V SOD1	49	10	cervical
9	M	FALS	A4V SOD1	55	21	thoracic and cervical
10	M	FALS	A4V SOD1	47	15	thoracic
11	M	FALS	N139K SOD1	50	74	cervical
12	M	FALS	C9orf72	56	24	lumbar
