# Supplementary material for: S-acylation of SOD1, CCS, and a stable SOD1-CCS heterodimer in human spinal cords from ALS and non-ALS subjects
Source: Sci Rep. 2017 Jan 25;7:41141. doi: 10.1038/srep41141 (PMC5264640; doi:10.1038/srep41141)
Supplement: Supplementary Information [file srep41141-s1.pdf]

Supplemental Information

S-acylation of SOD1, CCS, and a stable SOD1-CCS heterodimer in human spinal cords from ALS and non-ALS subjects

Sarah E. Antinone[1*], Ghanashyam D. Ghadge[2], Lyle W. Ostrow[3], Raymond P. Roos[2], and William N. Green[1*]

[1] University of Chicago, Department of Neurobiology, Chicago, 60637, USA
[2] University of Chicago, Department of Neurology, Chicago, 60637, USA
[3] Johns Hopkins University, Department of Neurology, Baltimore, 21205, USA
* wgreen@bsd.uchicago.edu or sarahantinone@gmail.com

Supplemental Figure S1

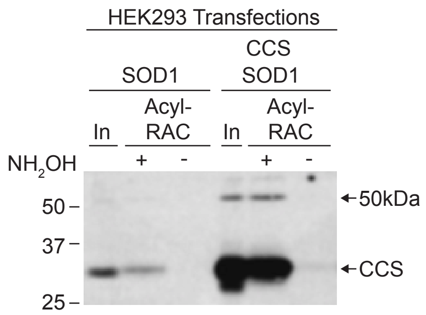

Supplemental Figure S1: Acyl-RAC detection of CCS and 50kDa band S-acylation. HEK293 cells were transiently transfected with either SOD1 alone or with both SOD1 and CCS. Equal amounts of total protein lysates were subjected to acyl-RAC analysis. 1% of input (In) protein, 100% of +NH2OH acyl-RAC bound protein, and 100% of –NH2OH acyl-RAC bound protein were analyzed by Western blotting with anti-SOD1 and anti-CCS antibodies.

Supplemental Figure S2

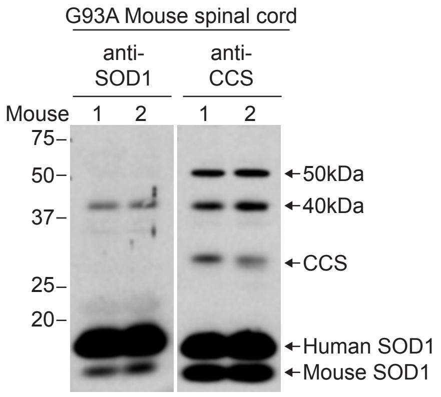

Supplemental Figure S2: Western blot detection of SOD1 and CCS in mouse spinal cords. Equal amounts of total protein lysates from spinal cords harvested from human G93A SOD1 transgenic mice (5.5 months old) were run on SDS-PAGE in duplicate and were analyzed by Western blotting with either an anti-SOD1 antibody or an anti-CCS antibody.

Supplemental Figure S3

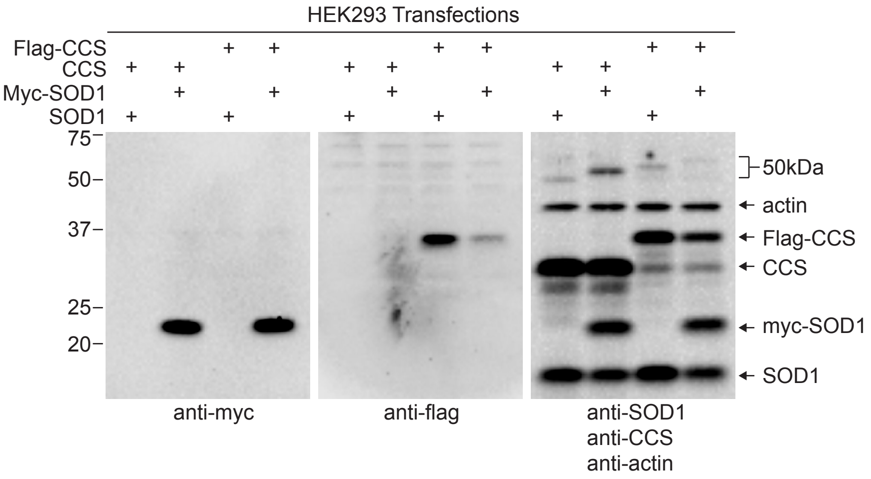

Supplemental Figure S3: The 50kDa band is a heterodimer of SOD1 and CCS. HEK293 cells were transiently transfected with the indicated untagged and tagged versions of SOD1 and CCS. Equal amounts of total protein from transfected cell lysates were analyzed by Western blotting with either an anti-myc antibody, an anti-flag antibody, or a combination of anti-SOD1, anti-CCS, and anti-actin antibodies.

Supplemental Figure S4

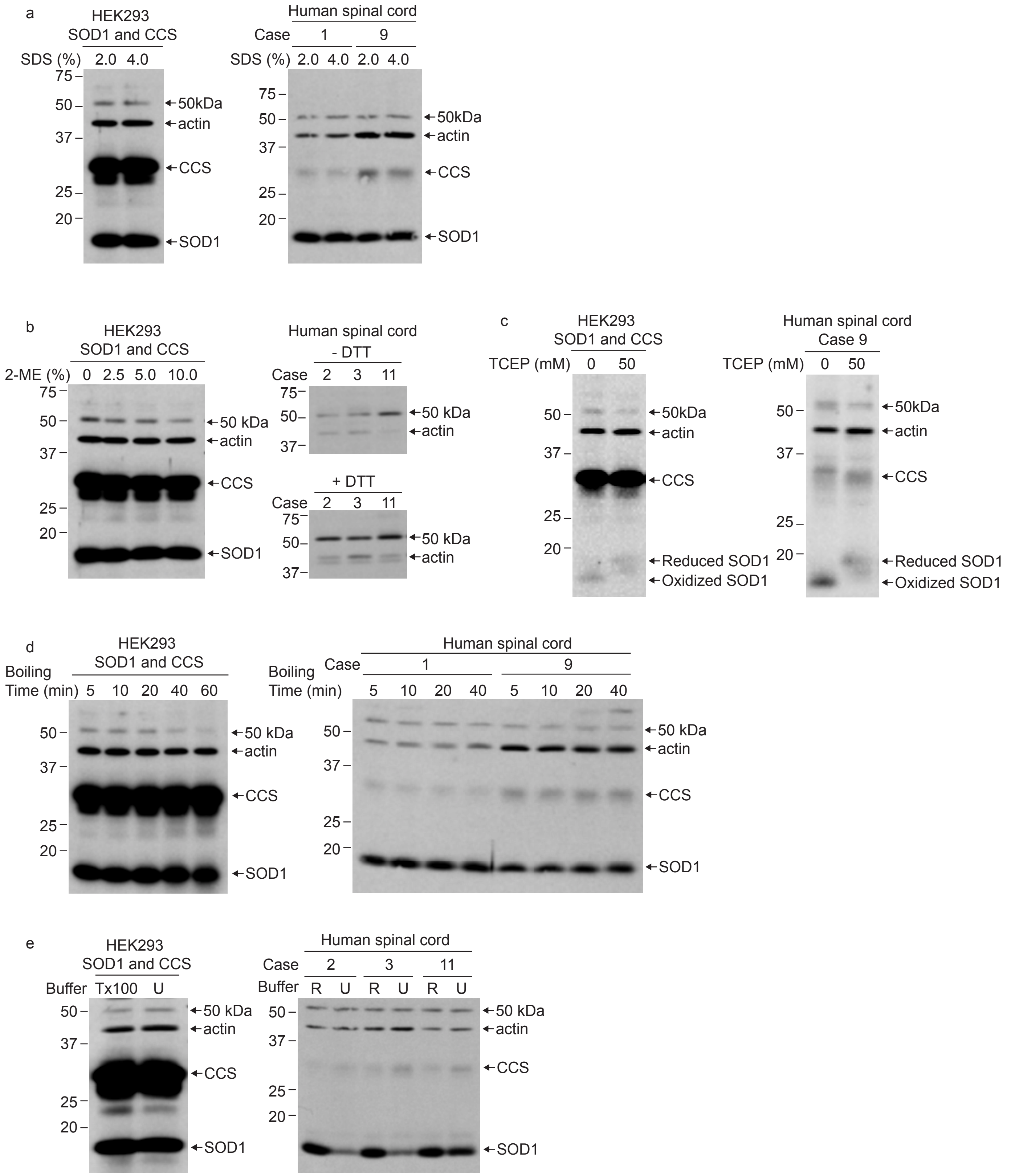

Supplemental Figure S4: Full-length Western blots from Fig. 4.

Supplemental Figure S5

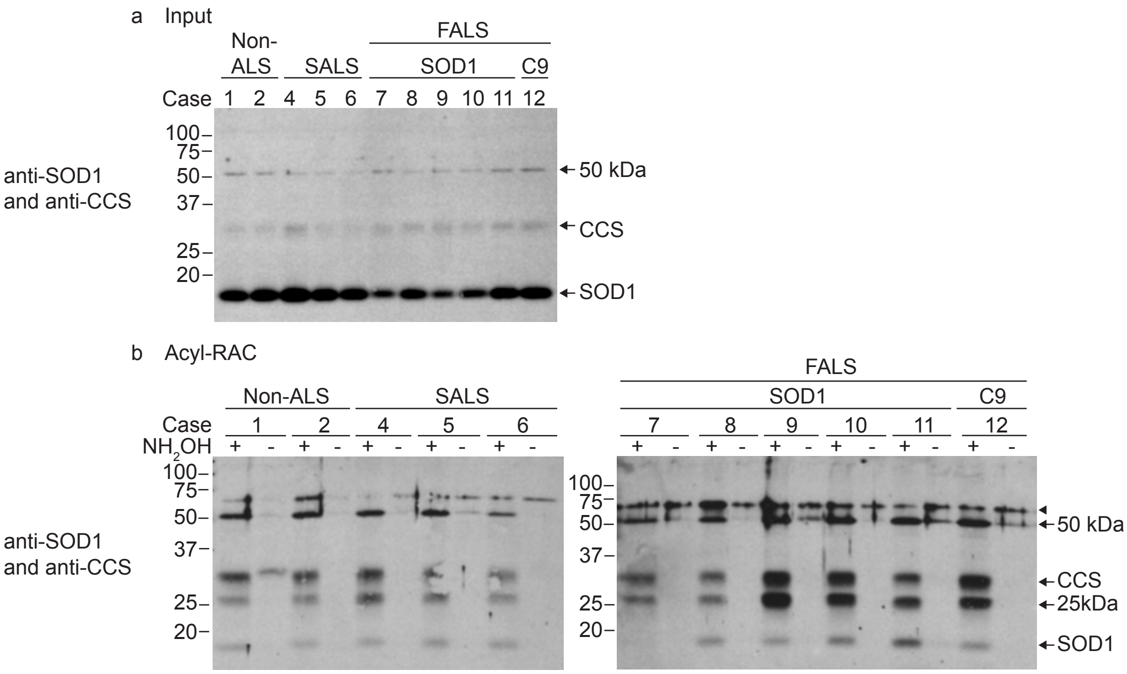

Supplemental Figure S5: Full-length Western blots from Fig. 5.

Supplemental Figure S6

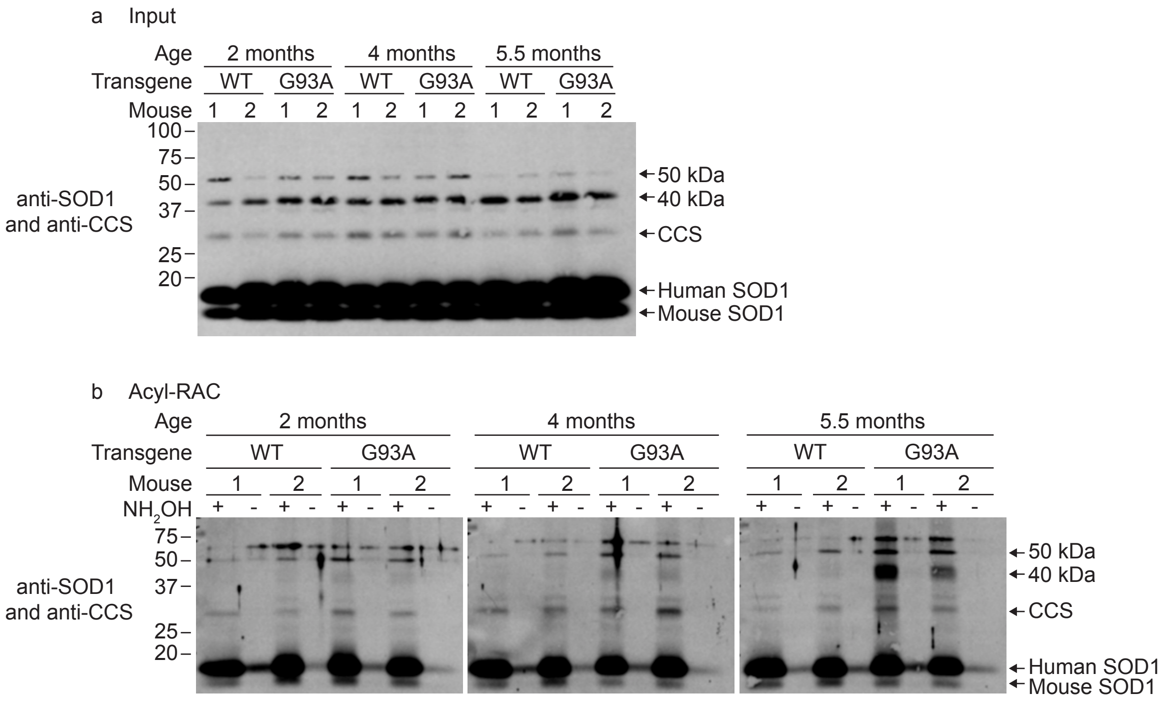

Supplemental Figure S6: Full-length Western blots from Fig. 6.
